# Co-parenting and Marital Satisfaction Predict Maternal Internalizing Problems When Expecting a Second Child

**DOI:** 10.1007/s12646-021-00620-z

**Published:** 2021-07-29

**Authors:** Sumeyra Yalcintas, Alison Pike

**Affiliations:** School of Psychology, University of Sussex, Brighton, BN1 9RH UK

**Keywords:** Perinatal mental health, Internalizing problems, Co-parenting, Marital satisfaction

## Abstract

**Purpose:**

Internalizing problems during and after pregnancy are important for parenting and child outcomes. The study aimed to understand correlates (i.e., marital satisfaction, co-parenting) of maternal internalizing problems during pregnancy with a second child.

**Method:**

We investigated levels of depression, anxiety, and stress symptoms of mothers in the third trimester of pregnancy with their second children. Fifty-one mothers and their firstborn children were visited in their homes and mothers completed questionnaires.

**Results:**

Results showed that co-parenting and marital satisfaction were related to internalizing outcomes. More specifically, co-parenting predicted depression  and stress  when controlling for marital satisfaction, whereas marital satisfaction predicted anxiety over co-parenting.

**Conclusion:**

The findings highlight the importance of studying prenatal internalizing problems differentially and can inform future intervention studies to prevent poor psychological outcomes.

## Introduction

The cost of perinatal mental health problems including depression and anxiety is around 8 billion pounds per year in the UK (Bauer et al., 2014). Pregnant mothers are monitored by general practitioners (GP) and/or midwifes, there are also some practices within mental health services specializing in mothers suffering from mental health issues. Pregnant women in the UK who are suffering from depression indicated emotional isolation and that research highlights the importance of increased midwifery support (Raymond, 2009). The purpose of the current study was to investigate the correlates of maternal internalizing problems in pregnancy with a second child. Based on family dynamics theory (Bowen, 1978) and current literature, we proposed two main predictors—co-parenting and marital satisfaction.

### Maternal Internalizing Problems

Overall, a good maternal mental health is linked with a good quality in parenting (Belsky, 1984; Belsky & Jaffee, 2015) and secure child attachments (Leerkes & Crockenberg, 2002) which are crucial for child development. Conversely, depressed mothers are more hostile (Lovejoy et al., 2000), demonstrate fewer sensitive behaviours (Feldman et al., 2009) towards their children, and have more negative interactions with their infants (Campbell et al., 1991). A large amount of research has been conducted on post-partum depression and its effects on infant development. For example, infants of depressed and anxious mothers demonstrated poor self-regulation (Feldman et al., 2009), and post-partum depression was related with infants’ language development at 12 months of age (Quevedo et al., 2012).

Pregnancy is also a critical stage for maternal well-being, which has received far less research attention. A systematic review suggested that mothers tend to report more depressive and anxiety-related symptoms (Bennett et al., 2004), and antenatal depression especially in the third trimester of pregnancy (Evans et al., 2001). In turn, maternal internalizing problems in pregnancy can lead to serious outcomes like underweight birth, mortality, and prematurity (Grote et al., 2010; Staneva et al., 2015). In addition, both depression and anxiety during pregnancy increase the risk of post-natal depression (Lee et al., 2007). Prenatal depression not only increased the risk for later depression but also increased the chances of post-delivery fear of childbirth (Bangma et al., 2020). Thus, considering the fact that about 20% of women suffer from prenatal depression (Bowen & Muhajarine, 2006), it is important to study the determinants of internalizing problems during pregnancy.

There is a good amount of literature devoted to understanding well-being during the transition to parenthood (Campbell et al., 1992; Hock et al., 1995; McDaniel et al., 2012). However, pregnancy with a second child can be more stressful for mothers, due to the needs of the firstborn. Research shows that second-time mothers have worse psychological adjustment compared to first-time mothers (Kojima et al., 2005). Mothers when they are pregnant with their second child, suffering from sleep deprivation, may struggle to regulate their executive functioning. Furthermore, having a firstborn aged 2–3 years old, in which most children develop self-regulation abilities (Thompson & Goodman, 2010) but also tend to show some problematic behaviours (Belsky et al., 1996) which is in turn linked with maternal depression and stress (Barker et al., 2011; Beck et al., 2004). Therefore, studying predictors of maternal depression, anxiety, and stress during this transition may be particularly informative.

Previous research has largely investigated single aspects of maternal internalizing problems (e.g., depression) or several aspects as a composite (e.g., depression, stress, and anxiety in combination). However, there are theoretical reasons to consider them differentially. For example, the Tripartite Model of Anxiety and Depression (Clark & Watson, 1991) explains the comorbidity of depression and anxiety by dividing them into physiological arousal, positive, and negative affect. In addition, studies investigating mothers during the prenatal and post-natal periods have demonstrated different findings for maternal anxiety and depression. For example, internalizing difficulties of children have been shown to be influenced by maternal depression but not by anxiety (Barker et al., 2011). Additionally, Lee and colleagues (2007) found that anxiety symptoms were more common than depressive symptoms in all stages of pregnancy. Yet the research on differentiation of these problems is limited. In the current study, we examined three different types of maternal internalizing problems, depression, anxiety, and stress.

### Co-parenting and Marital Satisfaction

The current study investigated co-parenting and marital satisfaction as predictors of maternal internalizing problems. Co-parenting can be defined as parents supporting each other in childcare, making decisions about the child together, as well as appraising each others’ parenting practices not undermining each others’ parenting, and it includes aspects such as co-parenting support, agreement about childcare, childcare division, endorsement, as well as conflict and undermining (Feinberg, 2003). Co-parenting plays an important role in the family system. Based on family dynamics theory, which emphasizes that members of a family are interdependent (Bowen, 1978), we suggested co-parenting as a predictor of maternal internalizing problems and is an important predictor of parenting, parental adjustment, and child development (Feinberg et al., 2007). Furthermore, several studies suggest that co-parenting support is related to lower levels of depression (O’hara & Swain, 1996), while co-parenting conflict and undermining are related to higher levels of depression (Solmeyer & Feinberg, 2011). Another study showed that low levels of co-parenting alliance increases parenting stress (Morrill et al., 2010). We know less about anxiety and specifically stress, as most studies focussed on parenting stress rather than general stress. Therefore, studying the symptoms of depression, anxiety, and stress in relation to co-parenting will contribute to the literature, especially in the stressful perinatal period.

Research on social support and family systems shows that low satisfaction with support is related to depressive and anxiety symptoms (Paarlberg et al., 1996). Importantly, the marital relationship is related with maternal mental health more so than other social relationships, including friends and family (Antonucci et al., 2001; Whisman et al., 2000). Marital satisfaction can be defined as how much a person is satisfied with their spouse/partner, and whether they are satisfied with their relationship and marriage as a whole domain. Not only does marital satisfaction show a gradual decrease in the transition to parenthood (Doss et al., 2009; Gottman & Notarius, 2000), it is also an important predictor of maternal internalizing problems (Kamp Dush et al., 2008; Whisman, 2001). Marital problems were related to more depression concurrently (Davila et al., 2003; Walker et al., 2013), as well as predicted depression one year later (Beach et al., 2003). Conversely, higher marital quality has been shown to be associated with better well-being over time (Proulx et al., 2007). Therefore, we also included marital satisfaction as a predictor of maternal internalizing problems.

### The Present Study

The study aims to understand maternal internalizing problems in pregnancy with a second child. Importantly, in this study, we examined three aspects of internalizing symptoms; depression, anxiety, and stress. Considering the likely correlation between co-parenting and marital satisfaction (Baril et al., 2007), we tested weighted contributions of marital satisfaction and co-parenting to each aspect of maternal internalizing problems.

## Method

### Participants and Recruitment

Participants comprised 51 mothers (*M*_age_ = 34.78 years; *SD*_*age*_ = 3.86 years) and their firstborn children (*M*_child age_ = 32.26 months; *SD*_*age*_ = 6.27) from the UK. Thirty of the children were boys and 22 were girls. Participants were recruited by emailing nurseries and via social media (Facebook groups) in the south of England, Sussex. Mothers were invited to participate in the third trimester of pregnancy (*M* = 33.43 weeks; *SD* = 4.45). All of the mothers were cohabitating with the father of the firstborn child; children were typically developing. 93.7% of the mothers reported having an undergraduate degree or higher. Forty-seven of the mothers reported their ethnicity as white. 86% reported having a job but only 6% had a full-time job.

### Procedure

Prior to the home visit, mothers were asked to complete an online questionnaire. The visits were conducted by two researchers and lasted 90–120 min. The researchers were trained extensively, had DBS checks and experience in the field. Then, one researcher interviewed the mothers and asked them to complete questionnaires, whereas the other researcher completed child tasks. Only mother-report questionnaires were used in the current study. Two of the mothers were living outside of the Sussex area. We could not conduct the home visits; however, they completed all of the questionnaires online.

### Ethical Issues

Ethical approval was gained from University of Sussex Sciences & Technology C-REC (ER/SY269/1) before recruitment commenced. British Psychological Society (BPS) ethical guidelines were followed throughout the study and mothers provided informed consent.

### Measures

*Maternal Internalizing Problems.* The Depression Anxiety Stress Scales (DASS-21; Crawford & Henry, 2003) were used to measure maternal internalizing problems. The 21-item questionnaire has three subscales, each with seven items. Mothers were asked to rate how much each statement was true for them over the past week on a 4-point scale (0 = *did not apply to me at all*, 3 = *applied to me very much or most of the time*). Scores for each subscale were summed and multiplied by 2. Sample items and reliabilities for depression were ‘I felt down-hearted and blue’ (Cronbach’s *α* = 0.80), for anxiety ‘I was worried about situations in which I might panic and make a fool of myself’ (Cronbach’s *α* = 0.77), and for stress ‘I tended to overreact to situations’ (Cronbach’s *α* = 0.79). The scale has great internal consistency and construct and convergent validity (Coker et al., 2018).

*Co-parenting Behaviour.* Co-parenting behaviour was assessed via the Brief Co-parenting Relationship Scale (CRS; Feinberg et al., 2012). This measure includes seven items; co-parenting support, agreement, undermining (reversed), closeness, endorsement, division of labour, and exposure to conflict (reversed). Sample items were ‘My partner and I have the same goals for our child’, ‘I feel close to my partner when I see him or her play with our child’, and ‘My partner is sensitive to our child’s feelings and needs.’ The response format is a 7-point scale (0 = *not true of us/never*, 6 = *very true of us/very often (several times a day*)). The scale has good reliability (Cronbach’s *α* = 0.76), stability, and construct validity.

*Marital Satisfaction.* The Kansas Marital Satisfaction Scale (Grover et al., 1984) was used to measure general marital satisfaction. The scale has three items. Mothers were asked how true each statement was for their feelings over the past month on a 5-point Likert scale (1 = *not at all*, 5 = *extremely*). A sample item is ‘How satisfied are you with your partner as a partner/spouse?’ The scale had a high reliability (Cronbach *α* =0. 94). The scale reported as having good internal consistency with having support for its validity (Sabatelli, 1988).

## Results

### Preliminary Results

Table 1 depicts the descriptive statistics and correlations among all study variables. Child’s gender and age were not related with maternal internalizing problems, so we did not control for these variables in the main analysis. Maternal age was correlated only with anxiety (*r* = −0.40, *p* < 0.05). Correlations among study variables were all in the expected direction and ranged in size from *r* = 0.25 to *r* = 0.72.

**Table 1:** Descriptive and correlations among study variables

Variable	1	2	3	4	5
1. Depression		.58**	.64*	− .45*	− .35*
2. Anxiety			.72**	−.29	−.42*
3. Stress				−.39*	−.25
4. Co-parenting					.65**
5.Marital satisfaction					
Mean (SD)	4.47 (4.50)	4.71 (5.78)	11.53 (6.13)	6.24 (.53)	4.32 (.62)
Range	0–16	0–24	0–28	4.64–7.0	3–5

^*^ *p* < .05, ** *p* < .001 *N* = 51

Maternal depression, anxiety, and stress were all interrelated, as expected. Similarly, marital satisfaction was related with co-parenting. Maternal depression was significantly correlated with co-parenting and marital satisfaction, more symptoms of depression were related with lower co-parenting and marital satisfaction. For anxiety, mothers’ age was significantly correlated with anxiety (*r* = −0.40, *p* < 0.05); the younger the mothers, the more anxiety-related symptoms they reported. Therefore, we conducted partial correlations to control maternal age. Results showed that the correlation between maternal anxiety and marital satisfaction was still significant after controlling for maternal age, more symptoms of anxiety were related with less marital satisfaction, and less co-parenting (*r* = −0.29, *p* = 0.049). Less co-parenting was associated with more stress-related symptoms, whereas stress was not related with marital satisfaction.

### Multiple Regression Analysis

In order to test independent contributions of marital satisfaction and co-parenting to the maternal internalizing symptoms, three multiple regression analyses were conducted. (see Table 2).

**Table 2:** Multiple regression models for three internalizing problems

	t	p	B	F	df	p	Adj R^*2*^
*Depression*							
Overall Model				6.41	48	.003	.18
Co-parenting	− 2.29	.026	− .39				
Marital Satisfaction	− .62	.540	− .10				
*Anxiety*							
Overall Model				5.89	44	.002	.24
Co-parenting	−.31	.755	−.05				
Marital Satisfaction	−2.20	.033	−.36				
Maternal age	−2.45	.018	−.32				
*Stress*							
Overall Model				4.29	48	.019	.12
Co-parenting	−2.25	.029	−.39				
Marital satisfaction	.02	.984	.003				

Standardized B values are reported. *N* = 51

#### Depression

The overall model was significant *F* (2, 48) = 6.41, *p* < 0.05, explaining 18% of the variation. Only, co-parenting significantly predicted depression (*B* = −0.39, *t* =  − 2.29, *p* < 0.05). Thus, with both marital satisfaction and co-parenting in the model, co-parenting was the only significant predictor.

#### Anxiety

Maternal age was correlated with anxiety so we included maternal age, as well as marital satisfaction and co-parenting, as a predictor. The overall model was significant *F* (3, 44) = 5.89, *p* < 0.01, explaining 24% of the variation. Marital satisfaction (*B* = −0.36, *t* = −2.20, *p* < 0.05) and maternal age (*B* = −0.32, *t* = −2.45, *p* < 0.05) were significant predictors. Thus, marital satisfaction was the strongest contributor to the model, followed by maternal age, whereas co-parenting was not a significant predictor.

#### Stress

The overall model was significant, *F* (2, 48) = 4.29, *p* < 0.05, explaining 12% of the variance. Co-parenting was the only significant predictor (*B* = −0.39, *t* = −2.25, *p* < 0.05).

## Discussion

The study focussed on predictors of maternal internalizing problems when expecting a second child. We tested two predictors and found that marital satisfaction and co-parenting were related with maternal internalizing problems consistent with the previous literature (Feinberg & Kan, 2008; Kamp Dush et al., 2008; Walker et al., 2013; Whisman, 2001). Finding that higher levels of marital satisfaction were related to less symptoms of depression and anxiety (Davila et al., 2003; Paarlberg et al., 1996; Walker et al., 2013), as well as co-parenting with depression and stress (Morrill et al., 2010; Solmeyer & Feinberg, 2011), replicated previous literature. However, we did not find any significant association between marital satisfaction and stress.

Considering the critical importance of maternal mental health during pregnancy (Bennett et al., 2004; Grote et al., 2010; Lee et al., 2007), we contributed to the literature by showing differentiated prediction of the three types of maternal internalizing problems. For depression and stress, only co-parenting was the independent predictor. However, for anxiety, it was only marital satisfaction that provided independent prediction. Thus, maternal depression and stress showed similar patterns, and this was different for anxiety. Thus, these findings highlighted the importance of studying internalizing problems differentially. The current literature on maternal internalizing problems tends to study internalizing problems as a monolithic construct rather than separating the symptoms of depression, anxiety, and stress. Considering the different associations, these symptoms should be studied in detail. In addition to showing that maternal internalizing problems should be studied differentially, we also demonstrated that marital satisfaction and co-parenting are not the same thing. Indeed, they are distinct and predicted different outcomes. Although we replicated that two concepts are inter-correlated (Baril et al., 2007). We also showed that co-parenting was a distinct predictor of depression and stress above and beyond marital satisfaction.

All in all, the findings can have implications for intervention studies and therapeutic settings. Previous intervention studies showed that it is possible to enhance mother’s and father’s co-parenting skills, and in turn, decrease symptoms of depression and anxiety (Feinberg & Kan, 2008). In addition, these same families showed decreased levels of parenting stress, 3.5 years after the intervention (Feinberg et al., 2010). The current study indicates that such an intervention study could prevent maternal depression, anxiety, and stress, if conducted in the perinatal stages. Interventions can focus to increase marital satisfaction and especially co-parenting, leading to better well-being outcomes. Partners can be taught several co-parenting strategies, like ‘discussing the best way to meet their child’s needs’ (Feinberg et al., 2012). The finding also can be useful when considering therapeutic settings. Especially, for the setting of couples therapy, partners can work on increasing satisfaction and co-parenting not only for maternal internalizing problems, but also to benefit child outcomes (Campbell et al., 1991; Feldman et al., 2009; Lovejoy et al., 2000). Crucially, co-parenting can be a more concrete and less vulnerable focus for intervention via a common focus, the child, partners can work to increase co-parenting.

### Limitations and Future Directions

A limitation of the study was the exclusive focus on mothers. Although fathers were asked to contribute, only 50% of the fathers completed the questionnaire. It would be ideal to make a composite score of co-parenting variable with higher contributions of fathers. However, we would argue that the outcome of interest was maternal internalizing problems, so mothers’ perspectives were essential. Additionally, considering the evidence from the literature suggesting that partner’s own marital satisfaction predicted depression of the spouse (Beach et al., 2003), paternal reports of marital satisfaction would also be vulnerable. To overcome this, future studies can encourage paternal contribution with preferably larger sample sizes. Another limitation is that our sample was largely white, and 93.7% of the sample had an undergraduate degree or higher, which might be an issue for generalizability of the findings. A majority of the mothers were also employed, which is associated with better well-being outcomes (Kleiverda et al., 1990). Therefore, future studies, with diverse educational and ethnical backgrounds, are needed.

Finally, the current study is coming from a longitudinal study’s first time point. The second time point, which we were collecting 4 months after the birth of the second child, has been disrupted because of the COVID-19 pandemic. Of course, to be able to predict maternal internalizing problems over time would be better than concurrent. Child behaviour problems tend to increase with the arrival of second born (Dunn et al., 1981; Gottlieb & Mendelson, 1990), and also there is a link between child behaviour problems and maternal internalizing problems, depressed mothers tend to have children with more behaviour problems than non-depressed mothers (Radke-Yarrow et al., 1992). Another possible predictor variable in a longitudinal study would be children temperament, considering mothers of children with difficult temperaments are considered at risk of depression and stress (Oddi et al., 2013; Pike et al., 2016). Therefore, with a longitudinal study, it would be possible to test the temporal ordering of associations.

## Data Availability

Not applicable (data are stored).
